# Aberrant adaptive immune response underlies genetic susceptibility to tuberculosis

**DOI:** 10.3389/fimmu.2024.1380971

**Published:** 2024-05-10

**Authors:** Anastasiia Tsareva, Pavel V. Shelyakin, Irina A. Shagina, Mikhail Yu. Myshkin, Ekaterina M. Merzlyak, Valeriia V. Kriukova, Alexander S. Apt, Irina A. Linge, Dmitriy M. Chudakov, Olga V. Britanova

**Affiliations:** ^1^ Precision Oncology Division, Boston Gene Laboratory, Waltham, MA, United States; ^2^ Institute of Translational Medicine, Pirogov Russian National Research Medical University, Moscow, Russia; ^3^ Abu Dhabi Stem Cells Center, Abu Dhabi, United Arab Emirates; ^4^ Department of Genomics of Adaptive Immunity, Shemyakin-Ovchinnikov Institute of Bioorganic Chemistry, Moscow, Russia; ^5^ Institute of Clinical Molecular Biology, Christian-Albrechts-University of Kiel, Kiel, Germany; ^6^ Laboratory for Immunogenetics, Central Tuberculosis Research Institute, Moscow, Russia; ^7^ Central European Institute of Technology, Masaryk University, Brno, Czechia

**Keywords:** TCR repertoire, tuberculosis, TB-susceptible mouse strain, CD4 + T cells, B cells, immunoglobulins, transcriptomic signatures

## Abstract

*Mycobacterium tuberculosis* (*Mtb*) remains a major threat worldwide, although only a fraction of infected individuals develops tuberculosis (TB). TB susceptibility is shaped by multiple genetic factors, and we performed comparative immunological analysis of two mouse strains to uncover relevant mechanisms underlying susceptibility and resistance. C57BL/6 mice are relatively TB-resistant, whereas I/St mice are prone to develop severe TB, partly due to the MHC-II allelic variant that shapes suboptimal CD4^+^ T cell receptor repertoire. We investigated the repertoires of lung-infiltrating helper T cells and B cells at the progressed stage in both strains. We found that lung CD4^+^ T cell repertoires of infected C57BL/6 but not I/St mice contained convergent TCR clusters with functionally confirmed *Mtb* specificity. Transcriptomic analysis revealed a more prominent Th1 signature in C57BL/6, and expression of pro-inflammatory IL-16 in I/St lung-infiltrating helper T cells. The two strains also showed distinct Th2 signatures. Furthermore, the humoral response of I/St mice was delayed, less focused, and dominated by IgG/IgM isotypes, whereas C57BL/6 mice generated more *Mtb* antigen-focused IgA response. We conclude that the inability of I/St mice to produce a timely and efficient anti-*Mtb* adaptive immune responses arises from a suboptimal helper T cell landscape that also impacts the humoral response, leading to diffuse inflammation and severe disease.

## Introduction

Tuberculosis (TB) remains the leading cause of mortality by a single infectious agent—according to the World Health Organization (WHO), 10.6 million people were diagnosed with TB in 2021, with 1.4 million deaths. The COVID-19 pandemic led to an increase in the number of undiagnosed and untreated TB cases, and thus a commensurate increase in TB transmission and the number of TB-associated deaths (1). TB is caused by *Mycobacterium tuberculosis* (*Mtb*), and predominantly develops in the lung after infection via the respiratory tract. Pulmonary inflammation subsequently arises as the result of complex interactions between *Mtb* and host immune cells (2). TB progression involves the recruitment of monocytes, neutrophils, and primed T and B cells to the lungs, culminating in the formation of dynamic lymphoid structures known as granulomata, which play a critical role in the anti-TB immune response (3). Experimentally, CD4^+^ T cells have been shown to be essential for establishing immunity against *Mtb* (4). The Th1-biased immune response, which activates macrophages via the major Th1 cytokine interferon (IFN-γ) is generally considered to be the most potent protective mechanism against TB (2, 5), although the frequency of specific IFN-γ-producing CD4^+^ T cells does not appear to correlate with TB protection (6). It should be noted that although roughly a quarter of the world’s population is estimated to have been infected with *Mtb*, only 5% of infected individuals will develop active disease while the other 95% remain asymptomatic, with the latter population being defined as having latent TB.

The factors that confer disease resistance in the population remain incompletely understood (1). Forward and reverse genetic approaches such as genome-wide association studies (GWAS) and the identification of cases of Mendelian susceptibility to mycobacterial disease in humans, as well as whole-genome mapping and knockout mutagenesis in mice, have identified a plethora of genetic loci and specific genes involved in mycobacterial infection control (7). Additionally, forward genetics or phenotype-based screening experiments in mice have demonstrated that independently established laboratory mouse strains differ by allelic variants at multiple loci that collectively regulate susceptibility to and severity of TB. These results collectively indicate that the mechanisms underlying TB infection control are polygenic (8–11). Among TB-susceptible inbred mice, two strains with exceptionally severe TB progression are particularly well characterized: C3HeB/FeJ (12) and I/StSnEgYCit (I/St) (9). These strains thus provide useful models for investigating immune factors involved in control of *Mtb* infection. A recent study of transcriptional signatures of *Mtb*-resistant [C57BL/6J (B6)] and -susceptible (C3HeB/FeJ) strains of mice attempted to draw parallels between mouse and human immune pathways involved in TB pathogenesis (13). This work determined that an increased type I IFN response—together with reduced T, B and NK cell signatures—was associated with greater TB susceptibility in C3HeB/FeJ mice. On the level of the immune response our previous studies showed, that I/St mice are much more TB-susceptible compared to the commonly-used B6 strain as assessed by all major severity phenotypes including survival time, cachexia progression, mycobacterial multiplication in organs, and lung pathology (14). It has also been demonstrated that early neutrophil influx leads to increased inflammatory infiltration by all major immune cell subsets, decreased type 1 cytokine production, and impaired anti-mycobacterial activity of lung macrophages in I/St mice (15, 16). These factors result in severe lung pathology, with necrotic granulomata surrounded by hypoxic zones (14, 17). We have also observed an earlier drop in lung B cell counts and more rapid disappearance of B cell follicles (BCFs) in TB-infected I/St compared to B6 mice, preceding diffuse pneumonia (18). Previous analysis has linked the TB-susceptibility of I/St mice to expression of the H2-A^j^ MHC-II allelic variant, whereas the H2-A^b^ allele expressed by B6 mice is associated with TB resistance (19). Furthermore, a comparison of mice bearing either the H2-A^b^ allele or H2-A^j^ allelic variant clearly showed profound shaping of the TCR repertoire, along with less efficient thymic selection of CD4^+^ T cells in the H2-Aj allelic context, and the authors of those studies proposed that the H2-Aj allele confers reduced capacity to present *Mtb* antigens to the CD4^+^ T cells that contribute to TB susceptibility (20). I/St counterparts also present a higher CD4/CD8 ratio than B6 mice, which might be explained by productive CD4^+^ thymic selection for the H2-E^j^ allele in I/St animals. Despite these insights, we still lack a detailed comparative picture of the immune response to *Mtb* in I/St and B6 mice at the molecular level, which could yield a deeper understanding of the differences between protective versus pathogenic immune reactions in the tuberculous lung.

In this work, we have attempted to uncover the molecular mechanisms of adaptive immune CD4^+^ T cells and B cells that underlie the multiple cellular and pathological differences observed in TB-susceptible versus -resistant mice. As shown in our previous studies, the genetic background of I/St mice provides a means for analyzing the adaptive immune response to mycobacterial infection that allows us to gain insight into the complexity of the mechanisms underlying the CD4^+^ T and B cell responses that contribute to TB susceptibility. We performed a systemic comparison of gene expression profiles from lung-infiltrating CD4^+^ T and B cells in TB-infected I/St and B6 mice. We then applied TCR repertoire analysis to elucidate basic differences between strains and identify *Mtb*-specific TCR clonotypes. In TB-resistant B6 mice, we found that pulmonary CD4^+^ T cells preferentially generate a convergent T cell response with a Th1 signature, while CD4^+^ T cells from TB-susceptible I/St mice developed inflammation without prominent sharing of antigen-specific TCRs. Additionally, we observed significant differences in the amount and isotype production of immunoglobulins between mouse strains. Our findings highlight the contribution of lung CD4^+^ T and B cells to the control of TB infection and shaping the overall immune response to TB. Furthermore, our results indicate that the manner of MHC-II presentation of *Mtb* antigens and interaction with CD4^+^ T cells may be one of the critical factors in shaping the immune response to TB and determining susceptibility or resistance.

## Results

As mentioned above, CD4^+^ T cells are the major effector lymphocytes in the response against mycobacteria. *Mtb*-specific CD4^+^ T cells begin to appear in infected lungs 2–3 weeks after low-dose aerosol challenge in mice (21). B cells also begin to infiltrate lung tissue and form BCFs, reaching a peak at 6–10 weeks post-infection; after that, the number of B cells and BCFs gradually decreases in susceptible I/St mice but persists longer during chronic infection in TB-resistant B6 mice (18). CD4^+^ T cells are both scattered in inflamed lung tissue and located within BCFs, where they interact with B cells and proliferate (22). To elucidate the signatures of the local adaptive immune response against *M. tuberculosis* in B6 and I/St mice, we isolated CD4^+^ T cells and B cells from mouse lungs at week 8 after low-dose aerosol infection with the virulent *Mtb* strain H37Rv and performed both transcriptomic analysis and TCR/BCR profiling. Differentially expressed genes in RNAseq data between B6 and I/St mice were evaluated with DESeq2 and shown on volcano plot (**Supplementary Figure S1**).

### Skewing of CD4^+^ transcriptomic signatures towards a pro-inflammatory profile in I/St mice

**Figure 1:**
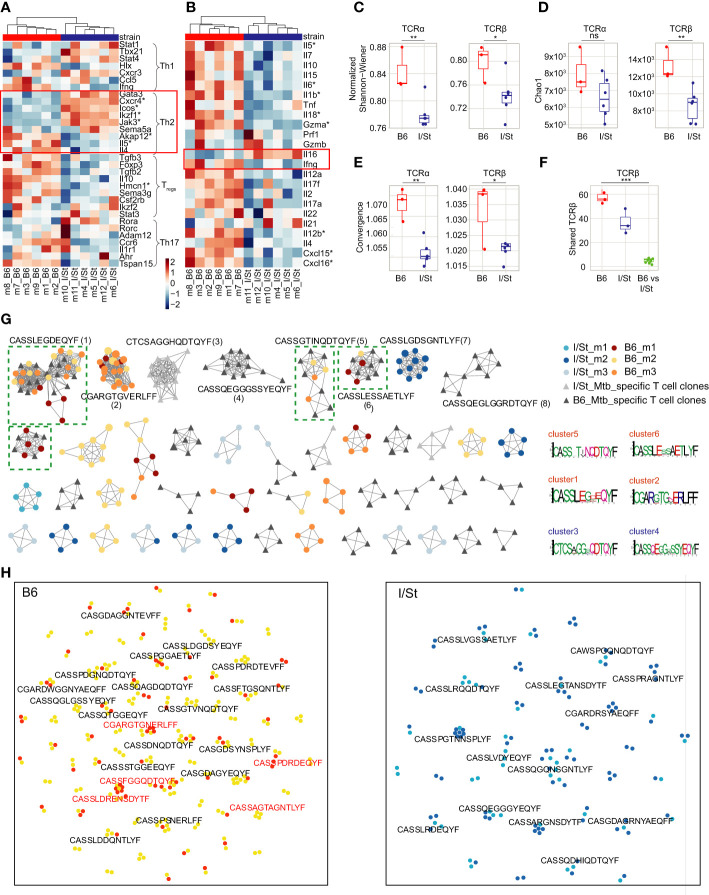
Analysis of gene expression patterns in CD4^+^ T cells. **(A, B)** Transcriptomic data revealed **(A)** upregulation of several genes associated with Th2 signatures (e.g*., Icos, Cxcr4, Ikaros Ikzf1*) in CD4^+^ T cells from I/St mice compared to B6, as well as **(B)** lower expression of certain Th2-associated cytokines (e.g., *Il4, Il5, Il6, Il10*). Red boxes show the gene signature of the Th2-type immune response. Stars denote genes that show statistically significant differential expression (adjusted p-values (Benjamin-Hochberg (BH) procedure) < 0.1 and log fold-change > 0.58). To identify enriched functional pathways within CD4^+^ population Ingenuity Pathway Analysis (IPA, Qiagen) and genes from (23) were used. **(C, D)** TCR repertoire profiling of CD4^+^ T cells from I/St and B6 mice. Downsampling to 12,000 TCRβ and 8,000 TCRα sequences in CD4^+^ T cells was performed three times independently, after which we plotted the average **(C)** Chao1 and **(D)** normalized Shannon-Wiener indices for the resampled repertoires. **(E)** Average convergence (i.e., mean number of unique CDR3 nucleotide sequences that code for the same amino acid sequence) for 12,000 TCRβ and 8,000 TCRα sequences in CD4^+^ T cells. **(F)** The number of shared TCRβ clonotypes (i.e., with identical amino acid sequence and V-segment usage) observed in pairwise comparison of the 1,000 most frequent clonotypes in CD4^+^ T cell repertoires from individual animals. All metrics in **(C–F)** were weighted by clonotype frequencies in the repertoire. N = 3 for B6 mice and 6 for I/St mice. Differences between mouse groups were tested using t-test adjusted with BH procedure p-values, and were labeled as ns (p > 0.05), *(p ≤ 0.05), **(p ≤ 0.01), or ***(p ≤ 0.001). **(G)** Clusters built from *Mtb*-associated TCRβ clonotypes identified separately for each mouse strain by ALICE. The 950 abundant TCRβ clonotypes were included from the CD4^+^ repertoires of B6 mice (N = 3; yellow, orange, brown), I/St mice (N = 3; light blue, deep blue, grey) and *in vitro*-expanded clonotypes for B6 and I/St T cells (black and grey, respectively) grown after stimulation *in vitro* with *Mtb* antigens. Logo plots of CDR3β motifs for the largest clusters are shown below. Clusters built from clonotypes including TCRs of *Mtb*-specific T cell clones labeled by green dashed square. **(H)** TCRNET evaluation of enrichment for full TCRβ repertoires of CD4^+^ T cells B6 (N = 6) and I/St (N = 6) mice. Orange and deep blue dots show shared clonotypes found at least two individual repertoires from B6 or I/St groups; yellow and light blue dots indicate private clonotypes. Red logos point out common clonotypes found among ALICE hits in B6 repertoires.

We isolated CD4^+^ T cells from lung tissue of B6 and I/St mice using anti-CD4 magnetic beads, with average purity of 95.2% as assessed by flow cytometry (**Supplementary Figure S2**; **Supplementary Table S1**). We assessed enriched gene pathways using Gene Set Enrichment Analysis (GSEA) incorporating gene sets from both M5 GO:biological process and M2 Wikipathways as these platforms are based on distinct gene sets. Up-regulated genes in B6 mice exhibited enrichment in the biological processes associated with Type II interferon (IFN-γ) and IL-17A signaling pathways as well as genes associated with humoral response (**Supplementary Figures S1C, D**). Transcriptomic analysis revealed upregulation of several genes associated with a Th2 signature [*e.g.*, *Icos, Cxcr4, Ikaros (Ikzf1)*] in I/St mice compared to B6 (**Figure 1**), although a subset of Th2-associated cytokine genes (*Il4, Il5, Il6, Il10*) was more highly expressed in B6 CD4^+^ cells. We confirmed changes in the expression of *Cxcr4, Icos, Ikzf1, Ifnγ, Il5*, and *Il4* in independent groups of mice by quantitative RT-PCR (**Supplementary Figure S3**). We also observed lower expression of several genes related with the regulatory T (T_reg_) cell transcriptomic program in I/St relative to B6, including *Foxp3, Il10, Hmcn1, Ikzf2,* and *Sema3g* (**Figures 1A, B**) (24). Interestingly, expression of IL-16, a CD4 ligand, was increased in I/St CD4^+^ T cells compared to B6 (**Figure 1B**), and this was confirmed by flow cytometry in independent experiment (**Supplementary Figure S4**). This cytokine has been reported to stimulate innate immune cells for production of proinflammatory cytokines such as tumor necrosis factor (TNF) and IL-6, and to act as a chemoattractant for eosinophils, monocytes, and T cells (25).

Genes linked to Th17 and Th1 cell profiles were heterogeneously expressed between B6 and I/St CD4^+^ T cells. Notably, *Ifnγ* expression was higher in TB-resistant B6 mice compared to I/St, that coincided with previous measurements of IFN-γ (19) and correlated with prior observations indicating a paramount role of IFN-γ-secreting CD4^+^ T cells in mounting an effective immune response against TB (26).

### TCR profiling reveals convergent antigen-specific response across B6 but not I/St mice

Interactions between antigen-MHC-II complexes and maturing CD4^+^ T cells during thymic and peripheral selection apparently shape TCR repertoire features at the level of naive T cells (20, 27, 28). This can subsequently alter the adaptive immune response against *Mtb* antigens in corresponding MHC contexts. We examined differences between lung-infiltrating CD4^+^ T cell repertoires of I/St mice carrying the MHC-II *H2-A^j^* and *H2-E^j^* allelic variants and B6 mice carrying only *H2-A^b^*.

We observed an opposite trend in reciprocal shifting of physicochemical characteristics in CDR3α and CDR3β repertoires between I/St and B6 mice (**Supplementary Figure S5**). Thus, the average value for the ‘strength’ of the interaction of CDR3β with pMHC was higher in I/St mice than in B6 mice, whereas for CDR3α, this parameter was the opposite (**Supplementary Figure S5С**). Strength is determined based on the frequency of strongly binding amino acid residues in the middle of CDR3. The comparison of the physicochemical characteristics of activated CD4^+^ T cell repertoire relative to the repertoires of B6 *H2-A^b^* and *H2-A^j^* naive CD4^+^ T cells (20) showed different pattern for CDR3α and CDR3β repertoires of I/St. This observation suggests the possibility of an influential role of *H2-E^j^* in shaping the repertoire of lung-infiltrating CD4+ T cells.

The normalized Shannon-Wiener index was significantly lower for TCRα and TCRβ repertoires of I/St mice compared to B6 (**Figure 1C**), and lung-infiltrating CD4^+^ T cells of I/St mice also tended to have lower TCR repertoire diversity compared to B6 mice (**Figure 1D**). The diversity was assessed using Chao1 which takes into account mainly singletons and doubletons while normalized Shannon-Wiener index assesses evenness of clonotype size distribution in repertoire. These results suggest accumulation of expanded T cell clones in TB-susceptible animals. At the same time, convergence was prominently higher in B6 repertoire than in I/St (**Figure 1E**). The combination of these parameters indicated that more clonotypes with different nucleotide sequences had identical sequences at the amino acid level in B6 mice. Notably, the repertoire of lung-infiltrating CD4^+^ T cells exhibited a significantly higher degree of pairwise overlap between B6 mice compared to that observed between I/St mice (**Figures 1E, F**), indicating a convergent antigen-specific response in the former.

To identify TCRs responding to *Mtb* antigens, we generated *Mtb*-specific T cell clones *in vitro* from isolated lymph nodes of B6 and I/St mice that were collected at least 21 days post-immunization with mycobacterial sonicate. After isolation, these T cells were co-cultured with the same mycobacterial sonicate followed by four cycles of expansion in the presence of splenic APC *in vitro* (see details in SI, **Supplementary Figure S6**). TCRβ repertoires from these antigen-expanded T cells contained 1,504 and 1,114 nucleotide clonotypes for B6 and I/St repertoires, respectively.

In order to identify TCRβ variants potentially selected for recognition to *Mtb*, we applied the ALICE algorithm, which allowed us to capture convergent TCR clonotypes involved in the immune response to specific antigens (29). We pooled the 950 largest clonotypes of B6 lung-infiltrating CD4^+^ T cells, I/St lung-infiltrating CD4^+^ T cells, antigen-specifically expanded B6 T cells and I/St T cells. The ALICE algorithm assumes that an ongoing immune response is typically driven by groups of convergent T cell clones with highly similar TCR sequences that expand upon antigen recall (30). ALICE further employs a V(D)J-recombination model to account for the presence of non-specific groups of frequently-generated TCR variants (31). ALICE identified 115 TCRβ cluster-associated clonotype hits in B6 mice, 80 hits in I/St mice, 158 hits in antigen-specifically expanded B6 T cells, and 48 hits in antigen-specifically expanded I/St T cells (**Figure 1G**). We next pooled ALICE hits obtained from each of the subsets and built clusters of TCRβ variants with a 1-amino acid (a.a.) mismatch allowed. Strikingly, the convergent clusters that included clonotypes from at least two mice almost exclusively comprised B6 hits. Of the 10 largest clusters, six belonged to B6 mice, and four of those (clusters 1, 5, 6, and 9) included clonotypes from the repertoires of antigen-specifically expanded B6 T cells, confirming their specificity to *Mtb* antigens. By contrast, only one I/St cluster (cluster 7) from a single mouse was observed in the top 10, and this was not confirmed by data from antigen-expanded I/St T cells. In general, TCRβ clonotypes from antigen-expanded I/St T cells did not overlap with the I/St clusters identified by ALICE in TCR repertoires of lung-infiltrating CD4^+^ T cells. These results clearly show a convergent, antigen-specific, public CD4^+^ T cell response in B6 but not in I/St mice.

As an additional test, we used a TCR neighbor enrichment test (TCRNET) (32) to extract homologous TCR sequences that are abundant in B6 CD4^+^ repertoires compared to I/St and *vice versa* (**Figure 1H**). We found more B6-enriched clusters than clusters originating from the I/St background, which is in line with our previous observations of high levels of convergence and overlap in B6 repertoires (**Figures 1E, F**; **Supplementary Figure S7**). The intersection of cluster-associated TCRβ clonotypes identified by ALICE and TCRNET showed 22 shared clonotypes in B6 repertoires, which included the members of the two largest B6 clusters (clusters 1 and 2) and only two shared clonotypes from I/St repertoires. Altogether, our cluster analysis suggests that B6 mice repeatedly produce lung infiltrating, *Mtb*-specific T cell clones of particular specificities that may allow them to control infection more efficiently, while the I/St response is far less focused.

### Different immunoglobulin isotypes are involved in immune response in *Mtb-*susceptible and *Mtb-*resistant mice

**Figure 2:**
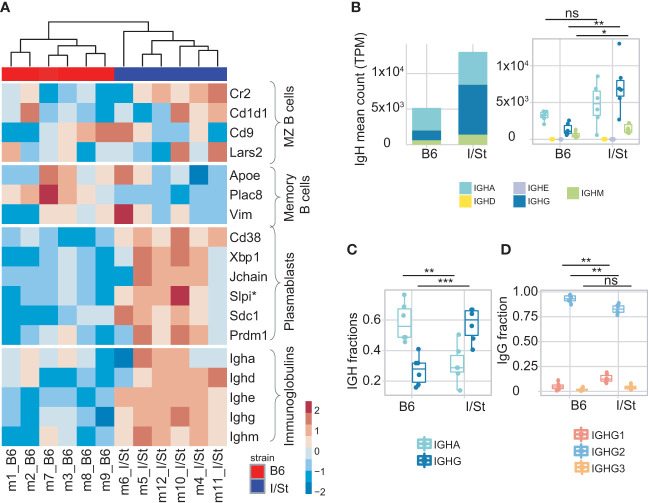
Analysis of B cells infiltrating lung. **(A)** Heatmap of normalized z-scores within each row of Deseq2 counts for the expression of a variety of signature set genes including IGH genes. **(B)** IGH counts extracted by MiXCR, normalized by total transcriptomic counts, and broken down by isotype. **(C)** Frequency of IgG and IgA in IGH repertoires. **(D)** Frequency of IgG1, IgG2, IgG3 within IgG repertoires in B6 and I/St mice (N = 6 of each strain). The difference between groups was assessed by t-test adjusted with BH procedure p-values, labeled as follows: ns (p > 0.05), *(p ≤ 0.05), **(p ≤ 0.01), and ***(p ≤ 0.001). Data on IGH isotypes and subclasses were extracted from RNA-Seq data of sorted B cells.

We isolated B cells from the lung tissue of each mouse with anti-CD19 magnetic beads. Flow cytometry analysis showed that the average purity of isolated B cells was 87% (**Supplementary Figures S2C, D**; **Supplementary Table S1**). We then performed bulk transcriptome profiling with extracted RNA from these cells. Using MiXCR (33), we extracted IGH CDR3 (BCR) repertoires from the RNA-seq data and assessed counts and fractions of isotypes in the samples from each mouse group. We found that expression levels of IgG and IgM were higher in transcriptomic data from I/St mice compared to B6 (**Figure 2A**) suggesting high proportion of Ig-producing cells. At the same time, the fraction of CD19^+^ B cells among all lung-infiltrating lymphocytes was lower in I/St mice (**Supplementary Figure S8**). Notably, IgG was the dominant Ig isotype in I/St repertoires, while IgA prevailed in the extracted repertoires of B6 mice (**Figures 2B, C**). IgG2 was the major IgG subclass in both strains (**Figure 2D**). Although the constant region of the IgG2 heavy chain is encoded by *Ighg2c* in B6 mice and by *Ighg2a* in I/St mice, the IgG2c and IgG2a proteins are functionally similar in mice and both are similar to human cytotoxic IgG1 (34, 35).

We observed a higher IgG1 fraction in the IGHG repertoire of I/St mice compared to B6 mice (**Figure 2D**), and this correlated with the measurement of serum *Mtb*-specific IgG1 concentrations using ELISA (**Supplementary Figure S9**). Of note, the murine IgG1 isotype is non-cytotoxic and similar to human IgG4 (36).

Consistent with our transcriptomic data, we detected notably higher secretion of antigen-specific IgM in serum from I/St mice relative to B6, which might indicate a delayed or distracted humoral response to *Mtb* (**Figure 2**; **Supplementary Figure S9**).

Focusing on the bulk gene expression profile of CD19^+^ B cells, we identified dominant transcriptomic signatures of plasmablasts versus memory B cells in I/St mice, which coincided with the high IGH counts we observed (**Figure 2A**). This suggested more earlier humoral immune response in the infected B6 mice than in I/St. Similar to our observations from CD4^+^ T cells, we found that expression of IFN-γ-inducible genes (*e.g., Ly6a, Ly6e*) was significantly decreased in CD19^+^ B cells from I/St mice, as was *Ifnγ* expression (**Supplementary Figure S10**). We also detected increased expression of *Cxcr5* and *Cxcr4* in I/St mice compared to B6 (**Supplementary Figure S10**). These genes encode receptor molecules for Cxcl13 and Cxcl12 chemokines, which are critical for B cell migration and the formation of B cell follicles and granulomata in TB (37, 38). Increased *Cxcr4* expression by B cells is indicative of enhanced migration of circulating B cells to pleural tissue (39). Altogether, our analysis of B cells in B6 and I/St mice revealed prominent inter-strain differences in gene expression profiles, reflecting a distinct mode of B cell response to *Mtb* in susceptible and resistant strains.

### Large IgA clonal lineages in repertoire indicates progressive immune response in *Mtb*-resistant B6 mice

**Figure 3:**
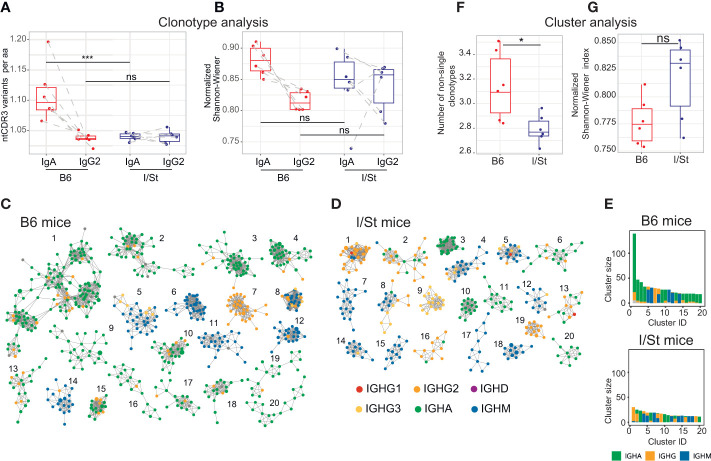
BCR repertoire profiling and clustering analysis of CD19^+^ B cells isolated from lung tissue of I/St and B6 mice. **(A)** Average convergence of CDR3 sequences, defined as the mean number of unique CDR3 nucleotide sequences divided by the mean number of unique CDR3 amino acid sequences. Convergence was calculated for the top 330 functional clonotypes from each isotype clonoset for each individual mouse. **(B)** Evenness of clonotype size distribution, as estimated by normalized Shannon-Wiener index, which was calculated for isotype clonosets that had been downsampled to 4,200 reads. Each data point was calculated from the mean of three independent downsampling iterations. Data points depicting IgA and IgG2 from the same mouse are linked with dotted lines. N = 6 for each mouse group. For **(A, B)** differences between groups were tested using the Wilcoxon test adjusted with BH procedure p-values, labeled as follows: ns (p > 0.05), *(p ≤ 0.05), ***(p ≤ 0.005). **(C, D)** The 20 biggest clusters of IGH clonotypes for **(C)** B6 and **(D)** I/St mice. For each group of mice, the top 3,000 clonotypes (by read count) were pooled from each IGH repertoire. 20 biggest clusters of IGH clonotypes for B6 **(C)** and I/St **(D)** mice are shown. Each cluster comprises the same V-segment with highly similar CDR3 amino acid sequences that exhibit no more than one mismatch. Each node represents a clonotype in the pooled repertoire, and nodes are colored by isotype according to the legend. **(E)** Sizes and isotype composition of top-20 IGH clusters, presented in panels **(C, D)**. **(F)** Average clonotype number in clusters containing two or more clonotypes. **(G)** Evenness of the cumulative clonotype size (i.e. number of reads) in each cluster, as estimated by normalized Shannon-Wiener index. The top 3,000 functional IGH clonotypes from each mouse were used to calculate values for panels **(E, F)**. Significance was calculated by Wilcoxon test.

We further assessed the number of unique CDR3 nucleotide sequences that encoded identical CDR3 amino acid sequences. In case of immunoglobulin profiling, this metrics reflects activity of somatic hypermutation. Notably, this metrics was significantly higher in IgA but not in IgG2 repertoires of B6 versus I/St mice (**Figures 3A, B**). Сlustering analysis revealed the formation of large, high-density IGH clusters (essentially representing B cell lineages) in B6 repertoires that predominantly incorporated IgA clonotypes (**Figure 3C**). In contrast, the IGH clonotypes of I/St mice formed significantly smaller clusters that consisted mainly of the IgM isotype (**Figures 3D–G**). These data indicate antigen-driven IgA evolution of the BCR repertoire in B6 mice, whereas B cells from I/St mice were most likely unable to generate a focused and protective immune response. Furthermore, the prevalence of IgM clusters might indicate a generally postponed immune response in I/St (**Figure 3D**).

## Discussion

We have examined the possible contribution of aberrant adaptive immunity to the more severe course of *Mtb* infection observed in I/St mice, focusing on lung-infiltrating CD4^+^ T cells and CD19^+^ B cells. Upon initial multiplication of mycobacteria in the lung tissue, dendritic cells (DC) deliver *Mtb*-antigens to the draining lymph nodes, present mycobacterial antigens in the context of MHC class II molecules, and activate T cells that subsequently migrate to the lungs. By eight weeks post-challenge, a full-blown adaptive immune response stabilizes *Mtb* proliferation in the lungs of mice from both strains, albeit at different levels (14). We selected this time point for our analysis because it reflects a stage of TB pathology that is relatively balanced between the exponential growth of the parasite during the initial and the terminal phase of the disease.

As shown previously, lung CD4^+^ T cells of I/St mice produce less IFN-γ than those from more TB-resistant mouse strains (15, 16, 19), and B cell follicles are less numerous in the lungs of these animals than in B6 mice (18). It is generally accepted that the tight balance of the Th1/Th2-type response is crucial for providing protective immunity against *Mtb* (3). Complementing previous data, we revealed several disturbances in the Th1 and Th2 signatures in the susceptible I/St strain versus resistant B6 mice. First, we observed upregulated expression of genes that promote the Th2 cell-fate program (*Gata, Icos, Cxcr4, Ikzf1*). Additionally, we revealed reduced expression of *Ifnγ* and associated genes (*Ly6a, Ly6e*) as well as decreased expression of T_reg_ gene signatures (*Foxp3, Il10, Sema3g, Tgfb2)*. Finally, we detected overexpression of *Il16*—among other cytokines—in I/St CD4^+^ T cells. This last observation was of particular interest, since the role of IL-16 in TB is poorly characterized. Enhanced IL-16 production has been shown to contribute to systemic inflammation by facilitating migration of Th1 and T_reg_ cells to the sites of inflammation (40). Additionally, IL-16 has been shown to inhibit phagolysosome formation and mediate subsequent bacterial replication (41), potentially promoting more severe disease. Emerging data presented evidence of elevated IL-16 levels in serum samples from active TB patients compared to those with latent TB. Furthermore, it highlighted that lower IL-16 levels are linked to less severe pathology and prolonged survival in a mouse TB model and confirmed the role of IL-16 in promoting *Mtb* intracellular survival by inhibiting phagosome maturation (42). We therefore believe that IL-16 might be one of the factors mediating inflammation in tuberculous I/St lungs.

The mode of antigen presentation by MHC molecules shapes the TCR repertoire during thymic selection (43, 44) and also triggers functional subset evolution upon antigen challenge. Thus, various HLA-DRB1 alleles modulate the balance between the Th1/Th2 cytokine responses to *Mtb* antigens (45). Studies in different human populations have revealed associations of HLA class II allelic variants with increased risks of developing symptomatic and severe TB (10, 46, 47). However, the underlying mechanisms of HLA association with TB susceptibility have remained elusive. Whilst studying functional CD4^+^ T cell subsets earlier, we obtained evidence that TCR rearrangements may determine major outcomes of cell activation by antigens (48). I/St mice express the *H2-A^j^* allele, which is associated with TB susceptibility and impaired CD4^+^ T cell selection. This mouse strain also carries the *H2-E^j^* allele, and this probably rescues a substantial portion of CD4^+^ T cells during thymic selection. However, it has also been shown experimentally that *Mtb* antigens could activate CD4^+^ T cells in the *H2-A^j^* context, but not with *H2-E^j^* (19). In the present work, we found that the TCR repertoires of lung-infiltrating CD4^+^ T cells in I/St mice had significantly lower diversity (*i.e.*, greater presence of clonal expansions). However, I/St TCR repertoires also exhibited lower convergence compared to B6 mice, which is defined by the formation of independently recombined but homologous or identical TCR variants that could recognize the same antigen (30). Thus, I/St lung-infiltrating CD4^+^ T cell repertoires contained evenly distributed and expanded T cell clones.

These findings raised the question of whether the observed TCR clonotypes were associated with a specific immune response induced by *Mtb*. Following the logic of repertoire convergence, we anticipated that TCRs of *Mtb*-specific lung CD4^+^ cells would form homologous clusters of highly-similar sequences that are shared within a given mouse strain, including *Mtb*-activated T cell clones that are enriched *in vitro* in the presence of *Mtb* antigens. Our clustering analysis indeed revealed large *Mtb*-specific TCR clusters in the repertoires of infected B6 mice, whereas such clusters were scarce in I/St mice. This suggests that excessive inflammation of the lung tissue near TB foci involves the extensive recruitment of non-*Mtb*-specific T cells, which may well exacerbate lung pathology rather than contribute to protective responses. It remains to be determined which inflammatory factors participate in this pathogenic pathway; IL-11 (49) and IL-16 (as found in this study) are possible candidates, since their expression is elevated in I/St compared to B6 mice. Regarding shifts from host-protective immune responses, it is worth mentioning an interesting recent cohort study that identified several TCR clusters recognizing different *Mtb* antigenic epitopes associated with either TB control or progression (50). The authors assumed that recognition of numerous *Mtb* proteins could defocus T cells away from essential responses to immune-dominant antigens. As such, the presence of decoy proteins could contribute to the observation that certain TCR specificities have no clear association with the outcome of control versus progression of infection. Together with the abundant presence of non-*Mtb*-specific T cell clones, these features of the overall T cell pool surrounding TB granulomata may explain the pathogenic T cell behavior observed in genetically TB-susceptible hosts.

We also observed distinct differences in the immunoglobulin response against *Mtb*. IgG2 appeared to be the major isotype both in B6 and I/St mice, but we found that more abundant total IgG and notable IgM production were characteristic features of the I/St strain. Interestingly, the IgA isotype was predominant among lung B cells from B6 mice. This was further supported by clustering analysis of IGH CDR3 regions, which showed that most clusters in the B6 repertoire consisted of IgA clonotypes, indicating active somatic hypermutation. In I/St mice, the IGH CDR3 clusters were smaller and formed preferentially by IgM, which corresponds with the higher *Mtb*-specific IgM response observed in the serum of I/St mice. This could reflect a delayed humoral response that contributes to TB susceptibility. Secretory IgA could interact with *Mtb* antigens and prevent epithelial cell infection in B6 animals, while IgG could promote infection in I/St animals as was shown experimentally (51). In line with our observations, IgA deficiency has been shown to lead to a delayed adaptive immune response to *Mtb* infection (52), and IgA-deficient mice ineffectively controlled infection against a background of reduced IFN-γ and TNF production by T cells (52, 53). The differences that we have observed in immunoglobulin profiles between mouse strains may be crucial for the further orchestration of an efficient response in resistant mice as well as the ineffectiveness of the anti-*Mtb* immune response in I/St mice. Accordingly, recent studies have shown that antigen-specific lung B cells could mediate *Mtb* control by directing the T follicular-like helper (Tfh) cell response (54), which plays an essential role in proper T cell localization for efficient macrophage activation during TB (55).

**Figure 4:**
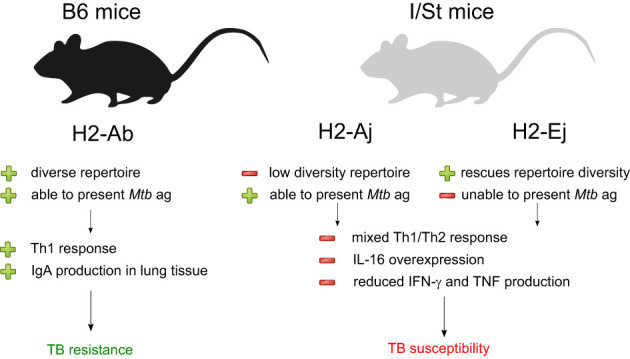
Summarization of the results of our analysis of *Mtb*-resistant and susceptible mouse strains. The presence of distinct MHC II allelic variants appears to have a strong influence on the nature and impact of the adaptive immune response to Mtb infection.

To summarize, our findings suggest that the presence of particular MHC-II alleles influences whether the adaptive immunity develops pro-inflammatory immune responses with mixed Th1/Th2 features, and this in turn shapes the antibody-mediated response to *Mtb* (**Figure 4**). The identification of these immune response patterns in mice could ultimately aid in diagnosing and assessing the severity of TB in humans. One recent study on TCR profiling in TB controllers and progressors proposed a strategy for vaccine development based on the identification of immunodominant antigens that cause efficient T cell responses in TB progressors, as determined by TCR repertoire cluster analysis (50). Our results suggest that such a strategy may be hindered by the inability of certain MHC molecules to present the necessary *Mtb* antigens in a manner that elicits an efficient T cell response.

## Materials and methods

### Laboratory animal model

10–12-week-old female mice from the I/StSnEgYCit (I/St) and C57BL/6JCit (B6) strains were used in this study. The animals were bred according to the guidelines from the Russian Ministry of Health #755 and US Office of Laboratory Animal Welfare (OLAW) Assurance #A5502-11. The Animal Facility of the Central Tuberculosis Research Institute (Moscow, Russia) provided water and food *ad libitum* under conventional breeding conditions. All experimental procedures were approved by the Institutional Animal Care and Use Committee (IACUC), protocols #1 and 3, on March 1, 2021.

### Infection

Mice were infected with ~10^2^ CFU of the virulent *Mtb* strain H37Rv (substrain Pasteur) using the Inhalation Exposure System (Glas-Col, Terre Haute, IN) according to a previously described protocol (56). At eight weeks post-infection, mice were euthanized by injection of thiopental (Biochemie GmbH, Vienna, Austria).

### Lung cell suspension preparation

Single-cell suspension samples were acquired separately from each mouse. Blood vessels were washed out by perfusion with 0.02% EDTA-PBS solution introduced through the right ventricle. The lungs were removed and sliced into 1–2-mm^3^ pieces. These were incubated in supplemented RPMI-1640 containing 200 U/ml collagenase and 50 U/ml DNase-I (Sigma-Aldrich, St Louis, MO) at 37°C for 90 min. The suspensions were washed three times in HBSS (Paneco, Russia) containing 2% fetal calf serum (FBS, General Electric, Boston, MA, USA) and antibiotics (Sigma, St. Louis, MO, USA). Next, the cells (50 x 10^6^ per dish) were incubated in 10 ml of medium (RPMI-1640, 10% FCS, 10 mM HEPES, 2 mM l-glutamine and antibiotics) in 90-mm-diameter cell culture-treated Petri dishes for 1 h at 37°C to achieve cell adhesion. Three repeated rounds of vigorous washing with warm antibiotic-free HBSS containing 2% FCS were performed to collect nonadherent cells. Two additional rounds of washing under the same conditions were then performed to get rid of residual medium. Cells then were resuspended in PBS, calculated, and proceeded for FACS analysis and cell sorting.

### B and T cell isolation procedure

Starting with 40 x 10^6^ cells per lung suspension sample, we respectively isolated B and T cells using anti-CD19 (Mojo, Bio-Legend) and anti-CD4 (MACS, Miltenyi Biotec) magnetic bead kits according to the manufacturers’ instructions. Sample purity was evaluated by flow cytometry. After sorting, cells were deposited in 350 ul RLT buffer (Qiagen) for cell lysis, RNA protection, and storage according to manufacture manual.

### Flow cytometry

Single-cell lung suspensions were analyzed by flow cytometry using FACS Cantu II machines (BD Biosciences). Detailed information on the procedure used for staining cells with antibodies can be found in **Supplementary Materials**.

### RNA isolation and сDNA synthesis for TCR repertoire profiling

RNA was isolated with the RNeasy Micro Kit (Qiagen), and the entire sample was used for the synthesis of first-strand cDNA with a mouse TCR profiling kit (MiLaboratories) according to the manufacturer’s protocol (see details in **Supplementary Materials**).

### RNA isolation and сDNA library preparation for bulk RNA sequencing

RNA was isolated with the RNeasy Micro Kit (Qiagen) from 5 x10^5^ cells. The SMART-Seq v4 Ultra Low Input RNA Kit (Takara) was used to prepare T and B cell cDNA libraries according to the manufacturer’s instructions. We used 5–10 ng of RNA per cDNA synthesis reaction, followed by tagmentation using the Nextera XT DNA library preparation kit (Illumina) and 10 cycles of PCR amplification according to manufacture recommendations. The amplified cDNA libraries were validated using the Agilent 2100 Bioanalyzer, pulled and purified using AMPure XP beads (Beckman Coulter). Final concentration of pulled cDNA library was measured with the QuBit dsDNA HS kit (ThermoFisher Scientific). Samples was sequenced on Illumina MiSeq and NextSeq 550, using 150 + 150 nt paired-end mode.

### Raw sequencing data analysis of bulk RNA-seq

The sequencing reads were adapter- and quality-trimmed, with a light quality cutoff of 20 Phred. For processed RNA-seq data, principal component analysis (PCA) and other analyses were used to check for batch effects and outliers. For adapter and quality trimming, fastp was used (57). FastQC and MultiQC were used to visualize raw and trimmed fastq files to access the read counts and quality metrics.

Raw RNA-seq data were aligned to the mouse genome using STAR. The FeatureCount tool was used to summarize the resulting bam files into raw read counts for QC (58). The R package DESeq2 was used for raw read count normalization (59). This tool was also used to perform differential gene expression analysis for B6- and I/St-derived T and B cells. Differentially-expressed genes were identified based on a fold-change of > 0.5 or < -0.5 and a false-discovery rate (FDR)-corrected p-value of < 0.1. All parameters were scaled using Z-score normalization. Visualization was performed using the programming language R and the ggplot software package.

### TCR and BCR repertoire analysis

Raw data analysis was performed with MiGEC (60), and using MiXCR (33). BCR heavy-chain repertoires were aligned to mouse BCR genes and assembled directly from raw RNA-seq data using MiXCR software (33). R was used to visualize the data and conduct statistical analysis using the Student’s t-test. The Benjamini-Hochberg (BH) procedure was used to correct all p-values for multiple comparisons with false discovery (at the rate ≤ 0.05). Clusterization of IGH chains and calculation of convergence (nucleotide per amino acid CDR3 sequence variants), Shannon–Wiener index, and diversity metrics were performed using in-house python script. Visualization of IGH clusters was performed using Cytoscape software.

All sequencing datasets have been deposited in the Sequence Read Archive (SRA) under BioProject accession number PRJNA1001295.

### CDR3 enrichment analysis with TCR-NET and ALICE

Neighborhood Enrichment Test (TCR-NET) analysis allowed us to find enriched CDR3 motifs that may encode antigen specificity. This pipeline is described in (61). Briefly, we pooled clonotypes assigned to TCRβ for each separate group of mice. We further calculated the number of equal TCRs that had identical or one amino acid substitution in CDR3 regions (*i.e.*, ‘amino acid neighbors’) in each pooled sample group. We selected clonotypes with increased clonotype numbers based on the following threshold: log10-normalized degree of connectivity fold-change > 0.5 and FDR-adjusted p < 0.005. We then constructed graphs based on these data in which nodes were considered to be connected in cases where the Hamming distance between “identical” amino acid sequences was equal to 1.

A similar search of CDR3 variants potentially responding to *Mtb* antigens was done with ALICE, which relies on the assumption that the antigen response simultaneously expands different clonotypes with similar CDR3 sequences (29). For each clonotype, ALICE assesses the number of amino acid neighbor clonotypes and compares it with the number expected from a neutral model of V(D)J recombination probabilities to account for the presence of non-specific groups of frequently-generated CDR3 variants (62). Clonotypes with significantly enriched neighborhood are considered as potentially responding to antigens. We applied ALICE as implemented in the package tcrgrapher (https://github.com/KseniaMIPT/tcrgrapher) to the 950 largest lung-infiltrating CD4^+^ T cell clonotypes from three B6 mice and three I/St mice (separately to each mouse), and to the 950 largest clonotypes of antigen-specifically expanded B6 and I/St T cells. To find similarities in response between samples, we pooled ALICE hits (FDR-adjusted p < 0.05) obtained for each of the subsets and built clusters of CDR3 variants with one amino acid mismatch allowed. Cytoscape was used to visualize clusters (63).

## Data availability statement

The datasets presented in this study can be found in online repositories. Raw sequencing reads are deposited in NCBI Sequence Read Archive under Bioproject accession number PRJNA1001295.

## Ethics statement

The animal studies were approved by the Russian Ministry of Health #755 and US Office of Laboratory Animal Welfare (OLAW) Assurance #A5502-11. All experimental procedures were approved by the Institutional Animal Care and Use Committee (IACUC), protocols #1 and 3, on March 1, 2021. The studies were conducted in accordance with the local legislation and institutional requirements. Written informed consent was obtained from the owners for the participation of their animals in this study.

## Author contributions

AT: Data curation, Formal analysis, Investigation, Project administration, Writing – original draft. PS: Data curation, Methodology, Visualization, Writing – original draft. IS: Investigation, Writing – original draft. MM: Data curation, Methodology, Visualization, Writing – original draft. EM: Investigation, Writing – original draft. VK: Data curation, Investigation, Writing – original draft. AA: Conceptualization, Writing – review & editing. IL: Conceptualization, Investigation, Project administration, Resources, Writing – original draft, Writing – review & editing. DC: Conceptualization, Writing – review & editing. OB: Data curation, Supervision, Writing – original draft, Writing – review & editing.
