# Supplementary material for: Aberrant adaptive immune response underlies genetic susceptibility to tuberculosis
Source: Front Immunol. 2024 May 10;15:1380971. doi: 10.3389/fimmu.2024.1380971 (PMC11116662; doi:10.3389/fimmu.2024.1380971)
Supplement: Supplementary file 1 [file DataSheet_1.pdf]

# Supporting Information for
## Aberrant adaptive immune response underlies genetic predisposition to tuberculosis.

Anastasiia Tsareva[1]*, Pavel V. Shelyakin [2,3] *, Irina A. Shagina[2,4] *, Mikhail Yu. Myshkin[4] *, Ekaterina M. Merzlyak[2,4], Valeriia V Kriukova[5], Alexander S. Apt [6], Irina A. Linge[6 #], Dmitriy M. Chudakov[2,3,4,7], Olga V. Britanova[2,4,5 #]

1 Precision Oncology Division, BostonGene Laboratory, Waltham, MA 02453, United States2-Institute of Translational Medicine, Pirogov Russian National Research Medical University, Moscow, 117997, Russia
2 Institute of Translational Medicine, Pirogov Russian National Research Medical University, Moscow, 117997, Russia
3 Abu Dhabi Stem Cells Center, Abu Dhabi, 4600, United Arab Emirates
4 Department of Genomics of Adaptive Immunity, Shemyakin-Ovchinnikov Institute of Bioorganic Chemistry, Moscow, 117997, Russia
5 Institute of Clinical Molecular Biology, Christian-Albrechts-University of Kiel, Kiel, Germany
6 Laboratory for Immunogenetics, Central Tuberculosis Research Institute, Moscow, 107564, Russia
7 Central European Institute of Technology, Masaryk University, Brno 62500, Czech Republic

* These authors contributed equally
[#] Corresponding authors: Olga V. Britanova, Irina A. Linge.

**Email:** olbritan@gmail.com; iralinge@gmail.com

**This PDF file includes:**

Supporting text
Figures S1 to S10
Tables S1-S2
SI References

**Supplementary Methods:**

**Flow cytometry.**
Single-cell lung suspensions were obtained from individual mice and cell phenotypes were analyzed by flow cytometry using the FACS CantuII machine (BDBiosciences) with subsequent labeled mAbs: anti-CD3-FITC (clone 145-2C11, BD Pharmigen), anti-CD4-BV421 (clone GK1.5), anti-CD4-PE (clone GK1.5), anti-CD4-PerCP (clone GK1.5), anti-CD8-APC (clone 53-5.8), anti-CD19-BV510 (clone 6D5), anti-CD19-PerCP (clone 6D5), anti-B220-AF488 (clone RA3-6B2), and anti-IgD-PE (clone 11-26c.2A, all Bio-Legend, Germany) were used. For intracellular staining of the lung cells, BD Fixation/Permeablization Kit (BD Biosciences) was used due to the manufacturers' recommendations. Briefly, after 16-17 hours of *in vitro* cultivation in the presence of mycobacterial antigens (10 mkg/ml), cells were harvested, stained first for surface antigens, then fixed with Cytofix/cytoperm buffer for 30 minutes and then stained for intracellular cytokines with rabbit anti-mouse IL16 pAB (Abcam) with subsequent donkey anti-rabbit secondary IgG-AF488 pAB.

**RNA isolation and Bulk RNA sequencing**
5x10$^5$ purified CD4$^+$ T and CD19$^+$ B cells from each mouse were supplemented with 350 mkl of RLT-buffer (Qiagen). RNA isolations were performed with RNeasy Micro Kit (Qiagen). SMART-Seq v4 Ultra Low Input RNA Kit (Clontech) was used for preparation T and B cells cDNA libraries according to the manufacturer's instructions. The overall amount of 5-10 ng of total RNA underwent reverse-transcription reaction. The cDNA is then amplified in PCR, tagmentaion was done using Nextera XT DNA library preparation kit (Illumina). The samples were mixed together equimolarly and purified using AMPure XP beads. Finally, the concentration of cDNA libraries was validated using the Agilent 2100 Bioanalyzer, and confirmed by RT-PCR with primers to Illumina universal oligoes.

**TCR repertoire profiling.**
Mixture of cDNA synthesis primers specific for TCR α- and β-chains with 12 μl of RNA was heated at 65°C for 2 minutes to denature RNA secondary structures. cDNA synthesis was performed 1h at 42C. After 1h

incubation, 1 µl of Uracyl DNA glycosylase (5 U/µl, NEB) was added, and additional incubation at 37°C for 40 minutes was performed to cleave residual NNoligos.Resulting cDNA was purified using 40ul AMPure XP beads (Beckman Coulter) and eluted in 10 or 30 ul of mQ according to manufacturer's instructions.Total cDNA was amplified in 21 cycles of PCR reaction for TCR α- and β-chains in a single tube. In the case of BCR heavy chain only 2,5 µl cDNA was used in 18 cycles of PCR amplification. For the second round of the PCR amplification 1 µl of the purified PCR product from 4.7 was used as a template. The PCR program was as described in 4.7 and 12 cycles were performed in this step. During this round, we added sample indexes and Illumina adapter sequences to both library ends. The PCR products were controlled using 2% agarose gel electrophoresis. The equal volumes of the final amplified and indexed cDNA libraries were mixed. The resulting mixture was purified with AMPure XP beads (Beckman Coulter) at 1 : 0.7 ratio (PCR product : beads). The concentration of the final libraries' mixture was measured with the QuBit dsDNA BR kit (ThermoFisher Scientific) and confirmed by RT-PCR with primers to Illumina universal adapters.

**Raw sequencing data analysis of bulk RNA-seq.**
After performed quality control we realized that the reads count was high for pseudogenes and the genes aligned to the HLA, TCRs, and immunoglobulins genes. These may be due to the absence of a reference genome for I/St mice and the genetically different MHC background for these mice strains. We additionally filtered out pseudogenes after alignment and grouped HLA, TCRs (in case of T cells), and immunoglobulins (in case of B cells) to reach more related to the immune response differences in differential expressed genes. Moreover, we observed some batch effects between two rounds of the experiment, but these did not affect significantly the obtained results.

**TCR and BCR repertoires post-analysis.**

All nonfunctional TCR and BCR clones, containing stop codons or a frameshift, were outfiltered at this step. For each sample, tables demonstrating TCRα or TCRβ or BCR heavy chain characteristics were obtained. The tables contain data on frequencies in the repertoires and V, (D), and J segments used in the generation of a corresponding clonotype.
The MiXCR output was converted to VDJtools format. To assess repertoire diversity characteristics (Shannon-Wiener normalized, Chao1 index), and CDR3 nucleotide length, data was downsampled to the minimal observed UMI count. Data without downsampling was used to calculate V-segment usage and physicochemical characteristics of the CDR3 region (an amino acid charge, strength, volume, polarity, hydrophobicity, Kidera factor 4). Obtained values were normalized to five according to the number of amino acids in the region of interest. All characteristics were weighted to clonotype frequencies in repertoires.

**Ig-ELISA**.
*M. tuberculosis* specific antibodies were quantified via ELISA by coating 96 well maxisorp immune plate (Thermo Scientific, Denmark) with 0,5 µg per well of mycobacterial cultural filtrate (CF) dissolved in 1 x PBS (Sigma-Aldrich, USA) at +4°C, overnight. Then wells were blocked with PBS containing 1% BSA for 1 h, room temperature (RT). Serum samples diluted to 1:200 in PBS containing 1% BSA (Calbiochem) were incubated for 1h, RT. Then HRP-conjugated goat anti-mouse IgM, IgG, IgG2a and IgG2c (JacksonImmunoResearch) were used as the detection antibody at 1:2000 dilution. For IgG1 detection biotinylated rat anti-mouse IgG1 (clone A85-1) were used as the detection antibody at 1:2000 dilution (1h, RT), with subsequent incubation with Streptavidin-HRP for 20 minutes, RT. Plates were developed using 3, 3', 5, 5'- tetramethylbenzidine hydrochloride (Sigma-Aldrich, USA) and optical density measured at 450 nm. All washes were performed with 1 x PBS, containing 0,05% Tween20.

**Stimulation/rest protocol for mycobacteria-specific T-cell clones and proliferation assays.** To prepare T-cell cones, cells from the popliteal lymph nodes of I/St and B6 mice, immunized into rear footpads with 10 µg/mouse of mycobacterial sonicate mixed 1:1 with incomplete Freund's adjuvant, were cultured as described previously (1) Briefly, 2 x $10^6$/ml immune cells (pooled from 3 mice of each strain) isolated on day 21 post-immunization were cultured in 24-well plates (Costar, Netherlands) in RPMI-1640 containing 10% FCS, 10 mM HEPES, 4 mM L-glutamine, 5 x $10^{-5}$M 2-ME, pyruvate, non-essential amino acids and antibiotics (all components–HiClone, Logan,UT, USA) for 14–16 days in the presence of 10 µg/ml mycobacterial sonicate. Live immune cells (>93% viability by trypan blue exclusion) were isolated by centrifugation at 2500 x g for 20 min at 20°C, on the Lympholyte M gradient (Cedarlane Labs, Ontario, Canada), washed twice and counted. The next stimulation cycle was accomplished by co-culturing 2 x $10^5$ isolated cells with mitomycin C-treated 1.5 x $10^6$ splenic APC in the presence of sonicate for another 14–16 days. These cycles were repeated 4 times and resulted in stable antigen-specific $CD4^+$ (>99% purity by flow cytometry) T-cell lines.
To assess antigen-specific proliferation, 15 x $10^3$ T cell clones of either B6 or I/St strain were co-cultured with $2{\times}10^5$ mitomycin C-treated corresponding splenic antigen-presenting cells (APC) in a 96-well flat-

bottom plate (Costar), at 37°C, 5% $CO_2$, in supplemented RPMI-1640 containing 10 µg/ml of *M.tuberculosis* sonicate or cultural filtrate (CF). Non stimulated wells served as controls. Triplicate cultures were pulsed with 0.5 µCi[$^3$H]-thymidine for the last 18h of 40h incubation. The label uptake was measured in a liquid scintillation counter (Wallac, Finland) after harvesting the well's contents onto fiberglass filters using a semi-automatic cell harvester (Scatron, Norway). Stimulation index was calculated as SI=cpm(+AG)/cpm(-AG).
**Antigens**. CF: Cultural filtrate is obtained by culturing M. tuberculosis as a biofilm on the surface of the protein-free Sauton's medium for 4 weeks, followed by precipitation of mycobacteria at 3,000g for 20 min, concentration of the protein content in the liquid phase using Amicon cell device, and bringing the final protein concentration to 1 mg/ml with sterile saline.
Sonicate: *M. tuberculosis*, disintegrated by ultrasonic (2).

| mice | $CD4^+$ T cells | $CD19^+$ B cells | mice | $CD4^+$ T cells | $CD19^+$ B cells |
|---|---|---|---|---|---|
| m1 | 94,50% | 90% | m4 | 95,40% | 90% |
| m2 | 94,40% | 90,10% | m5 | 96,80% | 84,60% |
| m3 | 94,60% | 91,30% | m6 | 95,70% | 87,10% |
| m7 | 95,80% | 84,60% | m10 | 97,80% | 85,60% |
| m8 | 94,90% | 85,80% | m11 | 97,60% | 89,70% |
| m9 | 97,20% | 84,60% | m12 | 96,60% | 88,90% |
| average | 95,23% | 87,73% | average | 96,65% | 87,65% |

**Table S1.** The cell phenotype was analyzed by flow cytometry using labeled mAbs. $CD4^+$ T cell purity analysis was performed using anti-CD3-FITC, anti-CD4-PE, anti-CD8-APC and anti-CD19-PerCP (Bio-Legend, Germany); B cell purity analysis was performed using anti-B220-AF488, anti-CD4-PerCP, anti-CD8-APC and anti-IgD-PE (Bio-Legend, Germany). Infiltrating $CD4^+$ T cells were isolated with the average purity of 85-90%. Infiltrating $CD19^+$ B cells were isolated with the average purity of 84-90%.

| Mouse id | Mouse strain | TCR chain | Number of clonotypes | Total number of UMI in clonotypes | Number of raw reads |
|---|---|---|---|---|---|
| m1 | B6 | alpha | 6970 | 27888 | 1249418 |
| m2 | B6 | alpha | 11436 | 43633 | 1681716 |
| m3 | B6 | alpha | 8970 | 41628 | 1167436 |
| m7 | B6 | alpha | 1137 | 2282 | 371711 |
| m8 | B6 | alpha | 707 | 1084 | 206758 |
| m9 | B6 | alpha | 712 | 1217 | 60633 |
| m1T | B6_cells | alpha | 1661 | 36492 | 1102972 |
| m10 | lst | alpha | 4443 | 24950 | 729585 |
| m11 | lst | alpha | 3725 | 10162 | 428628 |
| m12 | lst | alpha | 3091 | 10607 | 754149 |
| m4 | lst | alpha | 6462 | 39969 | 1516920 |
| m5 | lst | alpha | 6495 | 31962 | 711392 |
| m6 | lst | alpha | 6689 | 35481 | 1339569 |
| m2T | lst_cells | alpha | 984 | 13821 | 2367793 |
| m1 | B6 | beta | 7308 | 30711 | 1052425 |
| m2 | B6 | beta | 12885 | 52702 | 2008293 |
| m3 | B6 | beta | 10882 | 51567 | 2212126 |
| m7 | B6 | beta | 1135 | 2314 | 282135 |
| m8 | B6 | beta | 696 | 1150 | 139821 |
| m9 | B6 | beta | 773 | 1306 | 230412 |
| m1T | B6_cells | beta | 1504 | 36047 | 1101973 |
| m10 | lst | beta | 4595 | 29542 | 965282 |
| m11 | lst | beta | 4123 | 13419 | 566732 |
| m12 | lst | beta | 3143 | 12546 | 695009 |

| | | | | | |
|---|---|---|---|---|---|
| m4 | Ist | beta | 6964 | 62307 | 2401681 |
| m5 | Ist | beta | 7093 | 40352 | 1147189 |
| m6 | Ist | beta | 7269 | 46265 | 1539460 |
| m2T | Ist_cells | beta | 1114 | 19197 | 2840331 |

**Table S2.** Sequencing data on TCR repertoires of lung-infiltrating $CD4^+$ T cells and expanded T cell clones from *Mtb*-infected B6 and I/St mice.

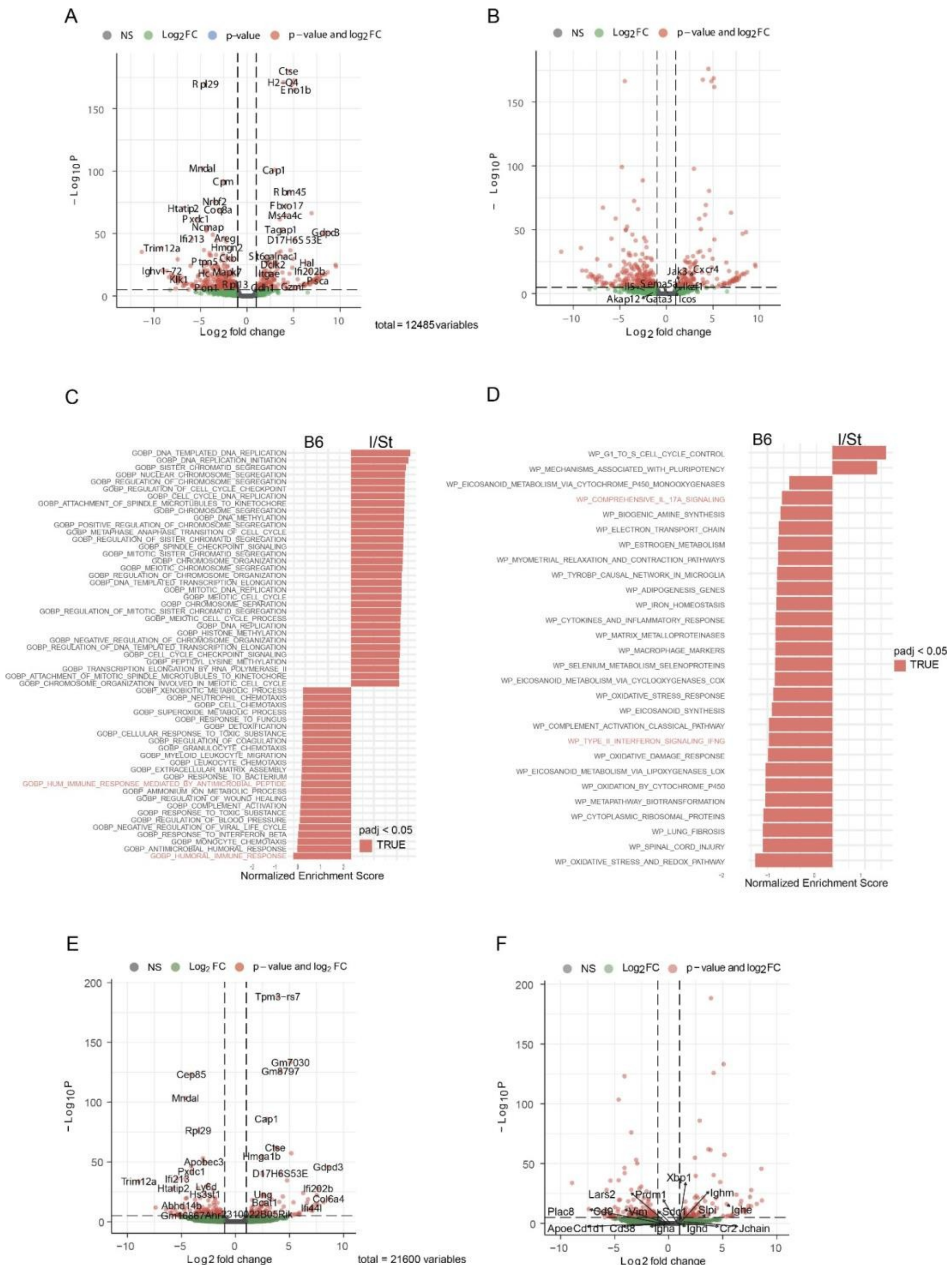


**Fig. S1**. Volcano plots of differentially expressed genes in CD4+ T cells and CD19+ B cells between I/St and B6 mice. (A, B) Volcano plots showing differentially expressed genes between B6 (left) and I/St (right) in CD4+ T cells. (A) All differently expressed genes are marked and (B) few subset marker genes are marked. (C, D) Gene set enrichment analysis (GSEA) of the transcriptomic data of I/St vs B6 CD4$^+$ T cells. Bars in red indicate significant enrichment at score > 2.1 for GO (C) and at score >1.3 for Wikipathways (D). Calculations were made for 6 mice per each group (E, F) Volcano plots showing differentially expressed genes between B6 (left) and I/St (right) in CD19$^+$ B cells. Hypothesis tested with DESeq2 was abs (LFC) > 0.58, adjusted p-value < 0.1.

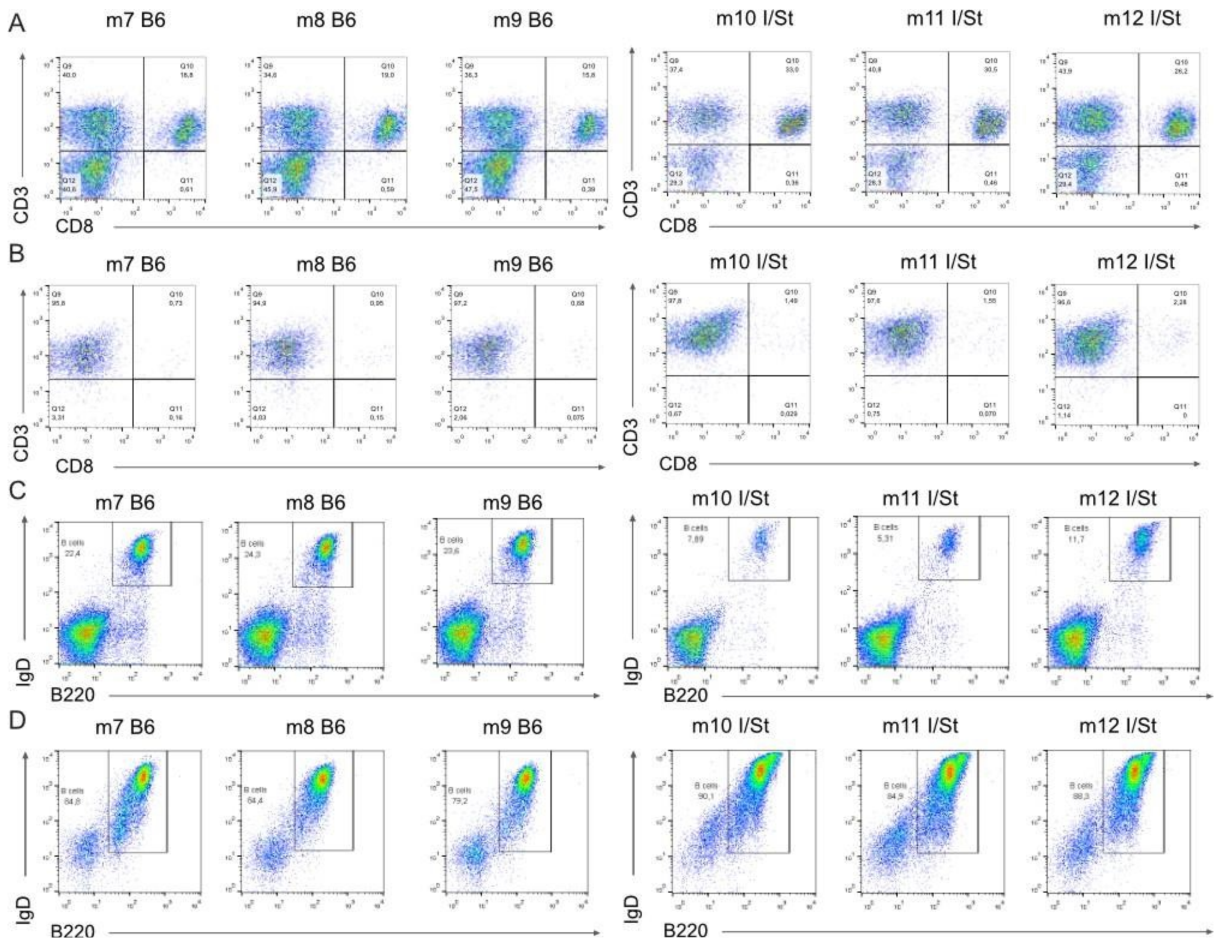


**Fig. S2.** T cells from each mouse separately were isolated with more than 95%. Flow cytometry analysis of B6 and I/St lung cells before (A) and after (B) sorting with magnetic beads. Results for gated lymphocytes are shown. B cells from each mouse separately were isolated with more than 87%. Flow cytometry analysis of B6 and I/St lung cells before (C) and after (D) sorting with magnetic beads. Results for gated lymphocytes are shown.

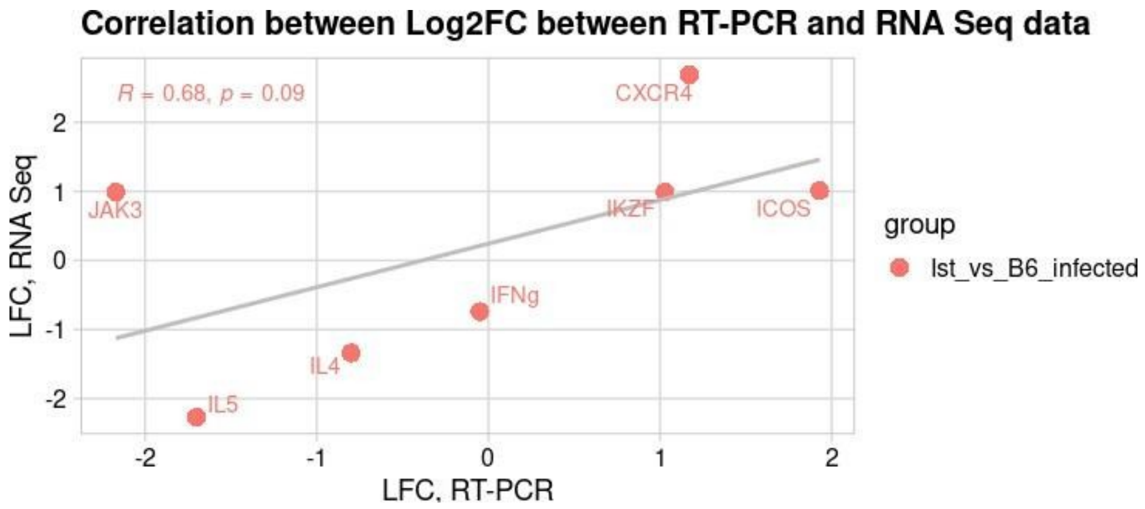


**Fig. S3.** Comparison of quantitative reverse transcriptase–polymerase chain reaction analysis of mRNA expression of the JAK3, IL5, IL4, IFN-γ, IKZF, CXCR4, ICOS genes found to be up- or down-regulated in the transcriptome of CD4$^{+}$ T cells purified from the B6 and I/St mice.

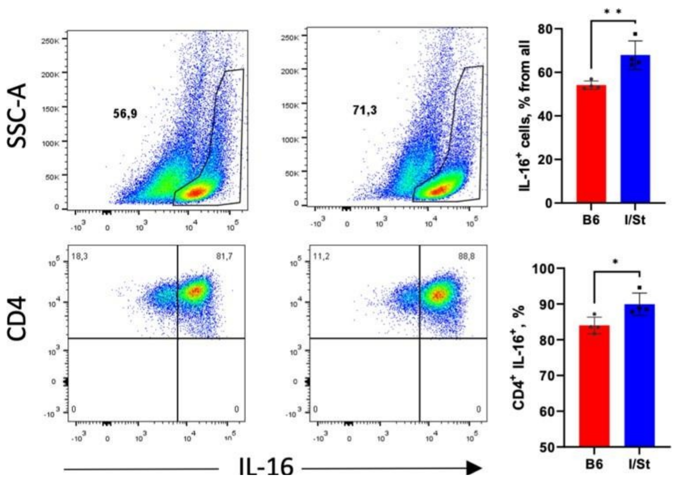


**Fig. S4**. Immunohistochemical staining for IL-16 of ex vivo lung cells, stimulated overnight with *Mtb* sonicate (anti-IL-16 rabbit pAB (Abcam) with subsequent staining with donkey aRbt-AF488). * $P<0.05$; ** $P<0.01$.

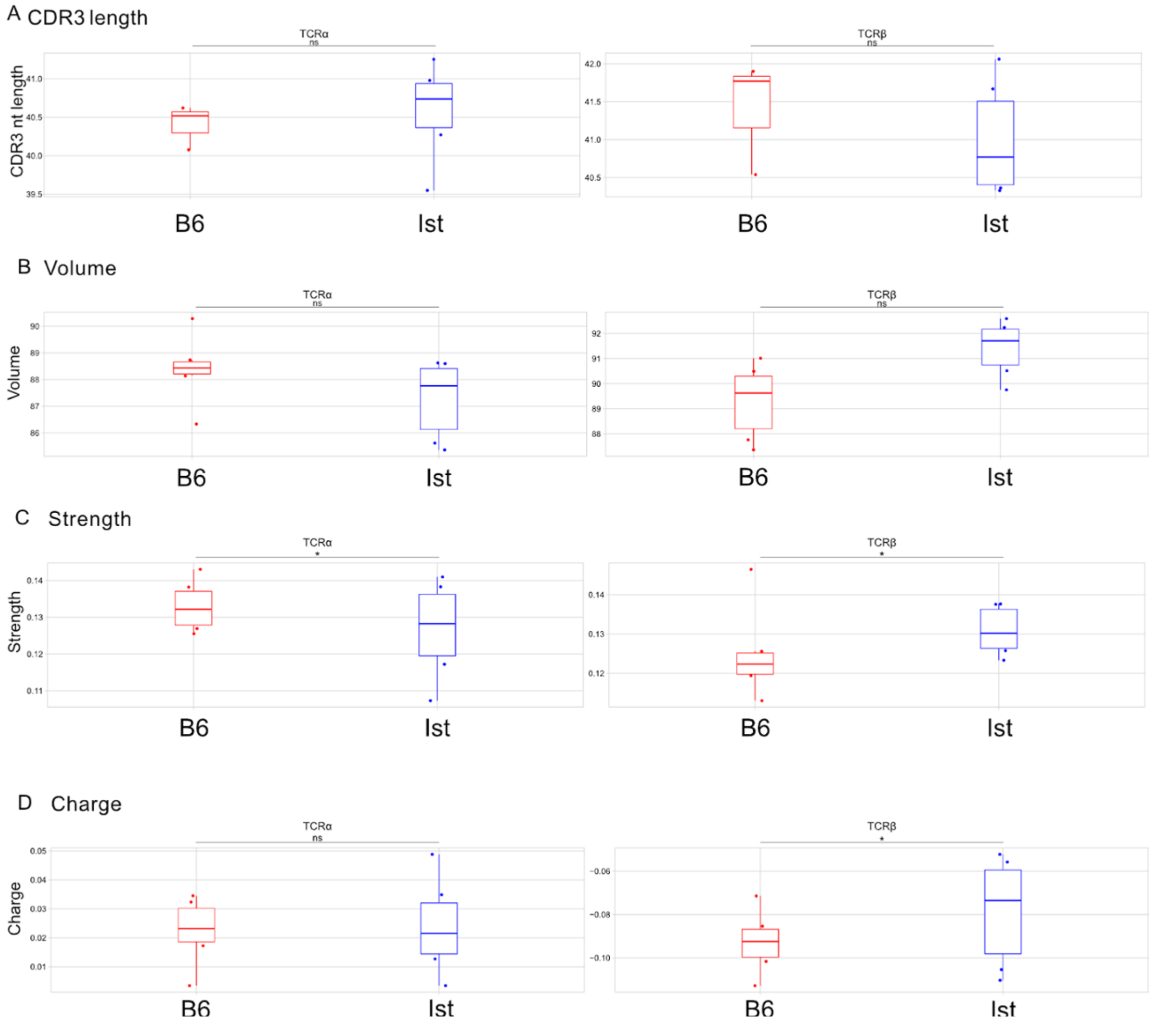


**Fig. S5.** Features of TCRα and TCRβ CDR3 repertoire. The average CDR3 nt length (A), the normalized average volume of an amino acid (B), the average number of strongly interacting amino acids (C), the average amino acid charge (D) within central CDR3 regions for 12,000 TCRβ, 8,000 TCRα in $CD4^+$ T cells. All metrics on the figure are weighed against clonotype frequencies in the repertoires. N = 4 mice for B6 group and N = 4 for mice for I/St group. The difference between groups was tested using t-test; adjusted by BH procedure p-values are labeled as ns $P > 0.05$, *$P \leq 0.05$, ** $P \leq 0.01$, *** $P \leq 0.001$.

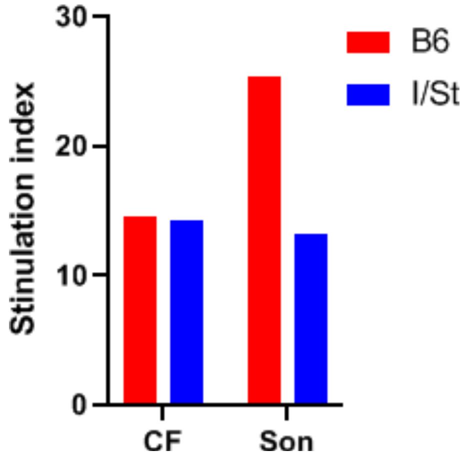


**Fig. S6.** In vitro proliferation of *Mtb*-specific polyclonal B6 and I/St T-cell lines in response to mycobacterial antigens, sonicate (Son) and cultural filtrate (CF), presented by MitC-treated splenic APC of B6 and I/St mice, respectively. Proliferation was estimated as [$^3$H]-thymidine uptake. Stimulation index = mean cpm of antigen-stimulated wells/ mean cpm of non-stimulated wells.

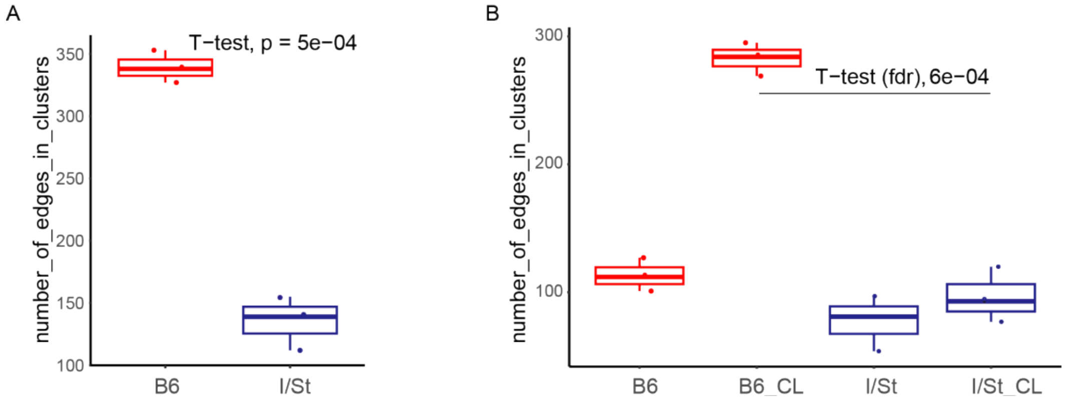


**Fig. S7**. (A) Number of edges within clusters from pooled top clonotypes from pair of mice of the same strain and from corresponding *Mtb*-specific T cell clones *in vitro*. Each dot corresponds to the value calculated for each group of top 950 (mice1) + 950 (mice2) + 950 (*Mtb*-specific T cell clones) clonotypes. Edge was drawn between pair of clonotypes if their CDR3 amino acid sequences were identical or contained no more than one mismatch. Number of edges reflects number and density of clusters. T-test was applied to assess statistical significance. (B) Number of edges within clusters from pooled top clonotypes for following pairs: 2 mice of the same strain (B6 or I/St) or one mouse and corresponding *Mtb*-specific T cell clones *in vitro* (B6_CL or I/St_CL). Each dot corresponds to the value calculated for top 950 clonotypes from each of two samples. Edge was drawn between pair of clonotypes if their CDR3 amino acid sequences were identical or contained no more than one mismatch. T test was applied with FDR. Labels on x axis: B6 - 2 B6 mice, I/St - 2 I/St mice, B6_CL - 1 B6 mouse and corresponding *Mtb*-specific T cell clones *in vitro*, I/St_CL - 1 I/St mouse and corresponding T cell clones.

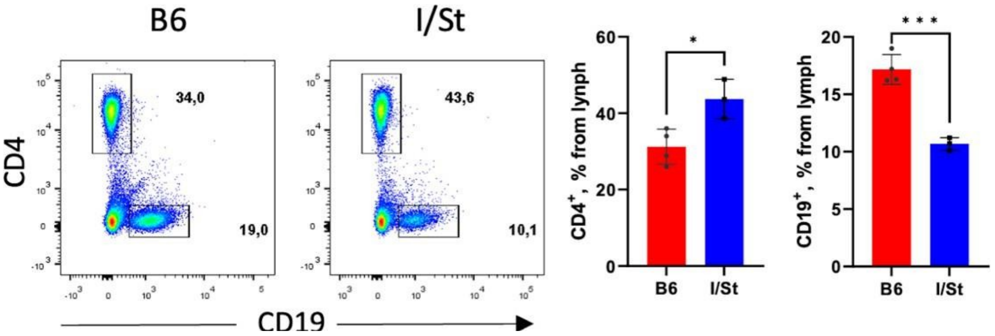


**Fig. S8.** Frequency of CD4⁺ T cells and B cells in the lungs of B6 and I/St mice at week 8 post low dose aerosol infection *with M. tuberculosis* H37Rv. (A) – Representative dot plots and frequency of CD19⁺ B- and CD4+ T cells measured by flow cytometry. The fraction of CD19⁺ and CD4+ cells were selected from the lymphocytes gated based on their FSC and SSC. The difference between the groups was tested using t-test, * $P$ <0.05; *** $P$ <0.001.

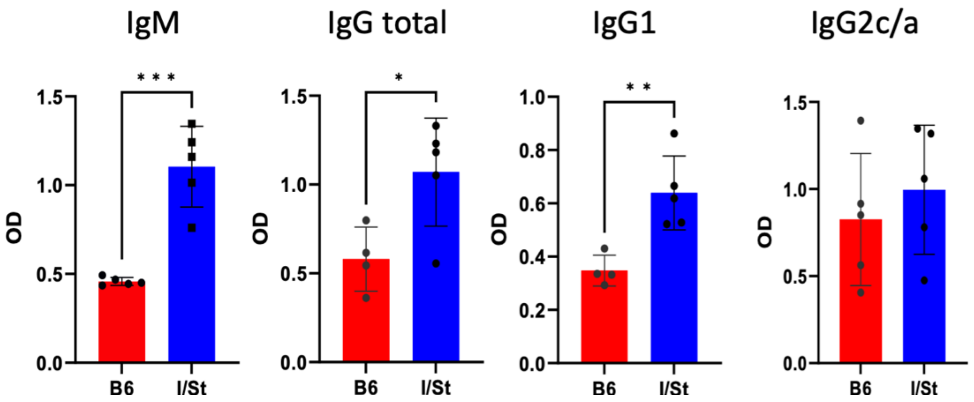


**Fig. S9.** *Mtb*-specific immunoglobulins measured by ELISA in serum of B6 and I/St mice at week 8 post low dose aerosol infection with *M. tuberculosis* H37Rv. The difference between the groups was tested using t-test, * $P < 0.05$; ** $P < 0.01$; *** $P < 0.001$.

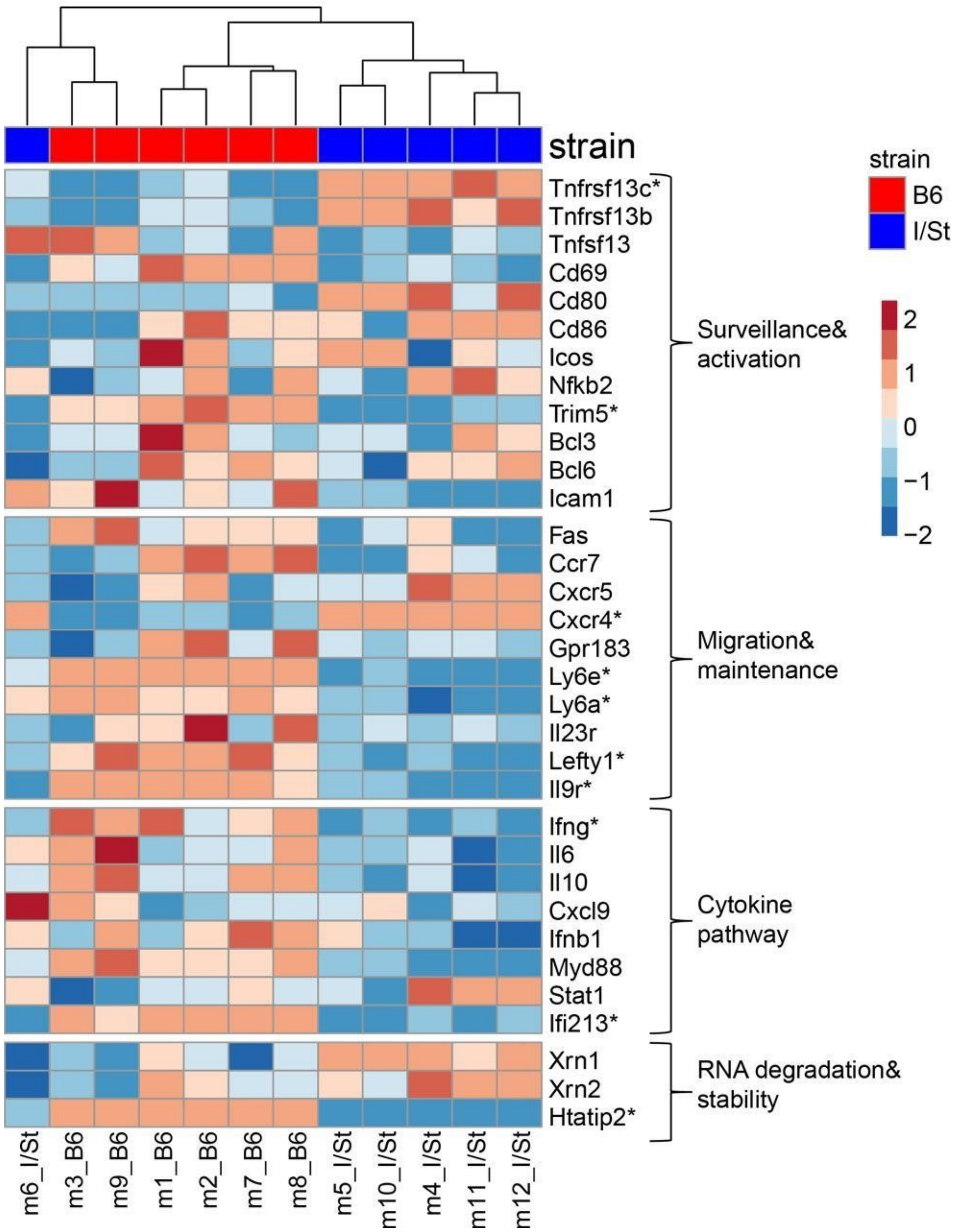


**Fig. S10.** Heatmap visualization of differentially expressed transcripts as analyzed by RNA-seq. Expression of B-cell-related genes across B6 and I/St mouse group. The genes are selected based on an adjusted p-value (Benjamin-Hochberg procedure) < 0.1 and a fold change >0.5.

**SI References**


1. Apt, A. S., V. G. Avdienko, B. V. Nikonenko, I. B. Kramnik, A. M. Moroz, and E. Skamene. 1993. “Distinct H-2 Complex Control of Mortality, and Immune Responses to Tuberculosis Infection in Virgin and BCG-Vaccinated Mice.” Clinical and Experimental Immunology 94 (2): 322–29.
2. Pichugin, A. V., S. V. Khaidukov, A. M. Moroz, and A. S. Apt. 1998. “Capacity of Murine T Cells to Retain Long-Term Responsiveness to Mycobacterial Antigens Is Controlled by the H-2
